# A Case of Epilepsy

**Published:** 1894-12

**Authors:** Isadore Gluck

**Affiliations:** Houston, Texas, Formerly, Omaha, Nebraska


For Texas Medical Journal.

### A CASE OF EPILEPSY.

BY DR. ISADORE GLUCK, HOUSTON, TEXAS, FORMERLY, OMAHA, NEBRASKA.

Read at meeting of Houston District Medical Society, October, 1894.

C. B., AGED 20, epileptic; father of marked neurotic temperament; dyspeptic, hypermetropic. Mother, healthy, of good physique, emetrope; four children, all living. Patient is the second eldest child. He resembles, in type, the father, and had his first attack of epilepsy in 1888. It was a cool summer evening. He was sitting in a tower of a large barn, reading a dime novel. Having a very emotional temperament, the topic of the novel being of an exciting nature, its perusal caused him extreme delight and emotion. Continuing his reading for sometime, he felt a slight sensation of numbness of his tongue, now and then a jerking. Soon his jaw stiffened. He still kept on reading, when at once everything became black before his eyes. He fell backwards, unconscious. He was taken home, bleeding from the mouth, in consequence of his bitten tongue, and was confined to bed for seven weeks, being unable to rise from his recumbent position without becoming dizzy. He recovered slowly. One year and a half after the attack, he commenced to read again, and kept it up until his second attack, which occurred August 26th, 1891, three years after the first.

It was again on an evening. He had been to the theatre, saw an exciting play, went home with the intention of finishing a dime novel. As he read, he felt all the pleasures and sorrows of the hero, as represented in the chapter he was reading. He experienced sensations varied in character and in intensity; he also experienced a numbness and jerking of his tongue, and when at the end of the chapter, scenes and conditions changed and interest commenced to vanish, the numbness and jerking of his tongue lessened. This activity and repose changing at intervals, numbness and jerking varying in intensity and duration, until, all at once, everything becoming black before his eyes, he lost consciousness, and a second attack ensued. He was found in this condition with his tongue bitten; he was put to bed and slept until morning, when he arose, feeling as well as usual. About a week later he ate some nuts, shortly before he retired, which caused prodromal manifestations, identical with those he had in the two former attacks. For five weeks he was unable to leave his bed, on account of dizziness when attempting to rise.

His condition remained the same, and was, at times, aggravated by the fear of an attack. The numbness of tongue and mouth has never left him, and becomes usually worse towards the close of the day. He never wanted to be alone, but must always have some living object in the room, be it a bird, a cat or a dog.

A year and a half previous to his first attack, he had a fall on his head. There was no injury at the time visible, nor is there any tenderness present. He also had a fall, which caused a deflected septum. His hearing was impaired. He was told by one of his attending physicians that he might expect an attack in any month, at the date of his last attack, and, towards the approach of that date of each month, he felt a great deal worse. He has been under treatment since his second attack, and some time ago he went East to consult a specialist on nervous diseases. The treatment consisted, as far as I can ascertain, of bromides. His last prescription was bromides and pepsin. At the time the patient was referred to me, ophthalmoscopic examination revealed fundus normal, slightly hyperæmic, hypermetropic, astigmatic. I ordered atropia. After using the same for four days, he complained of increasing numbness of tongue and mouth. I tested his vision. He accepted x0.50 cyl axis 90 with x0.50 sph. for each eye.

Although I was convinced that this was not all the latent hypermetropia, I discontinued the use of atropia, as its use aggravated the numbness, and, in addition, produced occasional twitchings of his arms. The test of his ocular muscles revealed exophoria, equal to 4 degrees, which, after return of accommodation, were normal.

Testing his left eye with the correcting glass, he read 20-xx. I then changed to the right, commencing again with letters at the top of the chart. When he reached the middle of the line designated by 20-xx, he stopped, turning his head from the chart, as one does when he wishes to avoid an unpleasant sight. I asked him why he did so, and, after a short pause, he answered, that he could not go on, as he could not articulate on account of the stiffness of his jaw. I naturally discontinued testing his eyes for that day, fearing, if I persisted, I might provoke an attack. Next day I reversed the procedure and began with the right eye. I asked him to read the line marked 20-xx, which he finished reading, when I changed to the left eye, and on the same line, but after he read two letters he was compelled to stop, on account of inability to articulate. Next I tested his ocular muscles for about one-half an hour, causing diplopia and fusion of images, binocular movements of the eye, without producing more than a slight dizziness, which befalls almost any individual undergoing a similar test. Now this patient, who is is peculiarly affected by the sight and reading of a few letters, is, otherwise, able to employ his eyes for hours, and perform the most delicate mechanical work. He writes with the same ease, but he is unable to read what he has written without producing all the manifestations of a beginning attack. These are indeed remarkable conditions. We have here an individual whose optical apparatus will perform its physiological function as a receiver and transmitter of sensations and objects without exciting any unusual phenomena. But let the character of images assume the shape of letters, the corresponding centers become peculiarly excited, and, let the source of excitement be persistent, it ends by upsetting the whole nervous apparatus, producing an explosion of nerve force equal to the magnitude and attempted control of the irritation.

Aside from the case being a unique one, it is highly instructive. After the first attack, it required one year and a half before he could resume reading. During that time he had no special treatment. Abstaining from reading, and avoiding all excitement, were the only therapeutics employed. He could read again without any ill effects. The question naturally arises, what occurred during and after the attack that made reading impossible, and what influence had the lapse of time and quietude upon his affection? In the light of our present knowledge of epilepsy, and from phenomena above cited, we are safe in assuming that after the fit the corresponding nerve centers became disorganized; that visual impressions were normally received and transmitted by the eye, but that its effect upon the centers of resistance was greater than the power of control. Also, that an irritation, or strain, upon a given center, in character the same as the primary, on the same route, requires a great deal less intensity and repetition to produce attacks. We also might add to this, that the intensity and repetition must be greater to produce same results, in proportion to the intervening time between attacks. In other words, as time causes repair and increases resistance, to produce the same effects, the irritation must be intensified. It took my patient a year and a half to have the damage repaired; time and rest being the principal restoratives. Although normal resistance was re-established, the source of irritation, the defective vision, remained, and thus, after reading again for one year and a half, a second attack was produced.

It was one year later he applied for treatment. When testing his eyes the first day, he could read a couple of dozen, or more, letters; next day he could only read a few, and an attack was imminent. Was the time intervening between the first and second days' test not sufficient for the reproduction of nerve fluid, or whatever it may be, that enters in the composition of central resistance? Or may it be that impressions form imprints upon successive layers, and that there had only been sufficient layers reproduced or regenerated during the twenty-four hours for the production of imprints for a few letters?

It is claimed by various writers on epilepsy that, in order to produce a second attack, the quality of disturbance must be synonymous, and must travel the same route. That dictum does not hold. I believe that, after the first attack, anything that will cause disturbance of vaso motor equilibrium, may cause its recurrence. My patient, one evening, ate some nuts, disarranging his stomach so that he had to go to bed. From that day on he had, every evening after supper (he being a hearty eater, and supper his principal meal), numbness of his tongue and stiffness of his jaw, together with dizziness and palor of face. Were not these epileptic attacks of lesser degree, and were they not caused by vaso motor disturbance? Was not this the cause in the case of Dr. Ranney, reported in the New York *Medical Journal*, June 10th, 1892, where the patient had at school thirty-four fits in twelve months, and, after correcting the defects of vision, he states that, "Over nine months had passed since an actual convulsion had occurred. . . . . . He imprudently used a lawn mower violently on a very warm day for several hours. As a consequence he was seized with one of his 'old time attacks,' having three severe convulsions and two light ones in the next forty-eight hours."

A good number of enthusiastic ophthalmologists tell us that there are many cases of epilepsy caused by defective vision, and that by correcting the visual and muscular defects the patient is cured. I should like to believe so, but I can not, because it is contrary to the experience of others, and to my own, which is not confined to this case alone, and it is also contrary to the findings of Schroeder, Van der Kolk, Brown-Sequard, Reynolds and Nothnagel. They agree, in substance, that the first seizure would have to be regarded as only a purely functional process, based as yet upon no change in the medulla oblongata or pons, but which, in itself, furnishes stimulus for the development of such, and Nothnagel states that after an epileptic attack changes in the substance of the medulla oblongata or pons have been demonstrated. And because of the occurrence of such a change in my patient, after the first attack had taken place, he was, in spite of the correction of his visual defect, unable to endure impressions of letters any more than he was before correction.

In the light of the above statements, the correction of refractive and muscular errors for the cure of epilepsy must be followed by a correction of the pathological condition, before a cure can be expected or pronounced.

---

For Texas Medical Journal.

